# Prevalence, Predictive Factors, and Characteristics of Osteoporosis in Hyperthyroid Patients

**DOI:** 10.1155/2018/3540256

**Published:** 2018-04-05

**Authors:** Ayotunde O. Ale, Anthonia O. Ogbera, Henry O. Ebili, Olusola L. Adeyemo, Taiwo O. Afe

**Affiliations:** ^1^Department of Medicine, Obafemi Awolowo College of Health Sciences, Olabisi Onabanjo University Teaching Hospital, Sagamu, Ogun State, Nigeria; ^2^Department of Medicine, College of Medicine, Lagos State University Teaching Hospital, Ikeja, Lagos State, Nigeria; ^3^Department of Morbid Anatomy and Histopathology, Obafemi Awolowo College of Health Sciences, Olabisi Onabanjo University Teaching Hospital, Sagamu, Ogun State, Nigeria; ^4^Obafemi Awolowo College of Health Sciences, Olabisi Onabanjo University, Ago-Iwoye, Ogun State, Nigeria

## Abstract

**Objective:**

The osteoporosis in thyroid disorder has the lowest report especially in sub-Saharan Africa. This study aims to determine the prevalence, predictive factors, and characteristics of osteoporosis in hyperthyroid patients.

**Method:**

Forty (40) hyperthyroid patients and healthy controls ages 21–50 years were recruited in this study. Questionnaires were administered to capture bio- and clinical data. Biochemical tests included blood, thyroid functions, intact parathyroid hormone, corrected calcium, and 25-hydroxyvitamin D tests. Bone mineral density (BMD) was also evaluated. Data were analyzed using the SPSS 21. A *p* value < 0.05 was regarded as significant.

**Results:**

Osteoporosis was observed in 18 (45%) of study subjects, 13 (72.2%) females and 5 (27.8%) males, respectively. The BMD of the hyperthyroid patients had a negative correlation with free triiodothyronine, FT3 (*r* = −0.49, *p* = 0.005), FT4 (*r* = −0.33, *p* = 0.009), corrected calcium (*r* = −0.31, *p* = 0.039), alkaline phosphatase (*r* = −0.53, *p* < 0.001), and osteocalcin (*r* = −0.61, *p* < 0.001). Conversely, a positive association with thyroid-stimulating hormone (TSH) (*r* = 0.54, *p* < 0.001) was observed. Multiple regression showed osteocalcin (*p* < 0.001) and TSH (*p* = 0.015) as independent predictors of osteoporosis.

**Conclusion:**

Thyrotoxicosis is a risk factor for osteoporosis occurrence, and we recommend routine screening for this bone disease in persons over 20 years old with this disorder.

## 1. Introduction

Thyroid diseases are among the commonest endocrine disorders worldwide [1]. Few studies had reported the prevalence of hyperthyroidism. In Africa, 2% and 1.6% prevalence rates were reported in South Africa and South West of Nigeria, respectively [2]. The estimated prevalence of overt hyperthyroidism in United States population ranges from 0.1% to 0.5% and is higher in females than males [3].

The common causes of hyperthyroidism in endocrinology clinics in Nigeria are Graves' disease, predominant in the young and toxic multinodular goitre, common in the middle-aged to elderly groups [2].

Although bone diseases are among the varied manifestation of untreated overt hyperthyroidism, they are usually underreported in sub-Saharan Africa.

In 1891, von Recklinghausen reported the first case of a young woman who died from hyperthyroidism with a feature of “worm-eaten appearance of long-bones.” Another study was reported by Plummer in 1920 with similar description [4]. Subsequent reports showed evidence that bone loss is common in overt hyperthyroidism.

Thyroid hormones are necessary for normal skeletal growth and development. The bone effect of hyperthyroidism is therefore characterized by accelerated bone turnover caused by direct stimulation of bones cells from high thyroid hormone concentrations and subsequently, this may result in loss of bone mass [5–7].

Hyperthyroidism increases bone turnover, with increases in both osteoclast and osteoblast activities [8]. As a result, the bone remodeling circle is shortened, although all phases of the cycle are not affected equally. The duration of the resorption phase is largely unaltered while the duration of the formation phase is reduced significantly. This leads to a failure to replace resorbed bone completely, resulting in net loss of about 10% of mineralized bone per cycle [9].

Early detection of osteoporosis and management of thyrotoxic state are therefore imperative to reduce the risk of fracture and improve the quality of life of patients with this disorder.

The paucity of epidemiological data and its defining characteristics on osteoporosis secondary to hyperthyroidism makes the exact disease burden difficult to ascertain in Nigeria. This study is therefore aimed at determining the prevalence of osteoporosis and evaluation of its clinical and biochemical correlates in hyperthyroid patients in order to create awareness on bone disease in hyperthyroidism.

## 2. Methods

This study was a prospective study spanning one-year period carried out in the Endocrinology and Metabolism Unit of the Department of Medicine, Lagos State University Teaching Hospital (LASUTH), Ikeja. Lagos is a state situated in the South West region of Nigeria. LASUTH is a state-owned tertiary health facility that receives referrals from other parts of Western Nigeria.

### 2.1. Subjects

The study population comprised hyperthyroid patients between ages 21 and 50 years undergoing treatment at the Endocrine Unit of LASUTH. This study was approved by the Research and Ethical Committee of LASUTH (reference number LREC/10/06/141); all participants gave informed consent before the commencement of the study.

Forty (40) patients, of age range 21–50 yrs, with active thyrotoxicosis and who had the Wayne index score > 19 in addition to biochemical parameters of hyperthyroidism (low TSH and high FT3 and FT4) and satisfied other inclusion criteria and gave consent were selected by systemic random sampling (every 2nd consecutive patient). A total of forty (40) age - and sex-matched apparently healthy controls were included in the study population.

Subjects with chronic medical disorders (such as diabetes mellitus, connective tissue disorder, and chronic liver disease), pregnant women, menopausal women, family history of fractures or history of multiple fractures since childhood, history of drug use (of estrogen, thiazide diuretic, calcium for management of osteoporosis, and vitamin D) in the last 12 months and the presence of proteinuria as well as significant alcohol intake (defined by 24gm per day), and smoking (20 packet per year) and caffeine intake were excluded from the study.

### 2.2. Clinical Assessment

Sociodemographic and other relevant clinical data were obtained using interviewer-administered questionnaires by specialized physicians. Anthropometric indices were determined using standard protocol, and body mass index was calculated. Objective assessment of thyrotoxic state was performed with the use of Wayne index score [10]. Also, proximal myopathy and hypocalcemia were assessed in line with operational guidelines [11, 12].

### 2.3. Laboratory Assessment

Quantitative assay of osteocalcin, serum 25-hydroxyvitamin D, and thyroid function tests (TSH, FT4, and FT3) was performed at Immunoassay Laboratories, the pioneer laboratories for enzyme-linked immunosorbent assay (ELISA) in Nigeria. The parathyroid hormone was carried out at Mecure Diagnostic Laboratory.

#### 2.3.1. Biochemical Analyses

Five milliliters of fasting venous blood were collected under the sterile condition for clinical chemistry and hormone analyses. Three milliliters of the sample was centrifuged and the sera were separated the same day and distributed in aliquots and stored in a freezer below −20°C. Two milliliters of blood was collected into heparin lithium bottles for the determination of serum calcium, phosphorus, creatinine, and alkaline phosphatase.

Early morning urine sample was also collected in the fasting state and used for the assessment of calcium, phosphorus, and creatinine.

Calcium was determined by timed-endpoint method [13]; serum albumin measurement was based on BCG (bromocresol green) albumin assay method [14]; inorganic phosphorus was determined using molybdate method [15]; alkaline phosphatase was determined using Hausamenetal method [16] and creatinine by modified Jaffe method [17].

#### 2.3.2. Hormonal Analyses

Quantitative determination of serum osteocalcin, 25-hydroxyvitamin D, TSH, FT4, and FT3 was performed using enzyme-linked immunosorbent assay (ELISA) method, while parathyroid hormone (PTH) was determined by the electrochemiluminescence immunoassay technique.

### 2.4. Bone Densitometry

All the recruited subjects were evaluated for bone mineral density (BMD) a week after their second appointment.

Clinical evaluation of BMD was performed using Dual-energy X-Ray Absorptiometry (DXA)-Lunar PIXI Number 50734 at the First Diagnostic Centre, Ikeja, Lagos.

### 2.5. Operational Definitions

1. Thyrotoxicosis was defined by clinical and/or biochemical evidence of hyperthyroidism: FT4 > 22.0 pmol/L, FT3 > 6.5 pmol/L, and TSH < 0.5 *μ*/U/ml. Overt hyperthyroidism was defined as Wayne score > 19 in addition to the biochemical pointers of thyrotoxicosis [10]. This index has been shown to have a diagnostic accuracy of 85% [10], sensitivity of 66–88%, and specificity of 92–99% when Wayne score < 20 [18].
2. Increased bone turnover was present if any of the following occurred in combination: serum osteocalcin > 25.3 ng/ml and/or total alkaline phosphate > 130 U/L (bone formation marker) and urinary calcium excretion > 4 mg/kg/day.
3. Osteoporosis was defined as normal biochemical levels of serum calcium phosphate and 25-hydroxyvitamin D with radiological evidence of osteoporosis either using the International Society of Clinical Densitometry (ISCD) criteria (established (severe) osteoporosis *z*-score < −2.0 with one or two osteoporotic fractures or osteoporosis by *z*-score < −2.0) [19]. The present study utilized the International Society of Clinical Densitometry criteria as the patients fall within the young age group.
4. Muscle power less than 3/5 by the medical research scale in the proximal group of muscles in the upper or lower limb was indicative of proximal myopathy [11] while sign of hypocalcemia was elicited by performing the Trousseau's sign [12].

### 2.6. Statistical Analyses

Data analysis was performed using Statistical Package for Social Science (SPSS for Windows version 21.0 SPSS Institute, Chicago, IL, USA). Quantitative (continuous) data were expressed as means and standard deviations (SD) while qualitative (categorical) data were reported as percentages. The Student's *t*-test was used to determine the association between quantitative data while the chi-square test was used for the determination of associations between qualitative data. Pearson's correlation coefficient was used to determine the extent of the relationship between the tested variables. Multiple regression analysis was used to determine the possible predictors of BMD.

## 3. Results

### 3.1. Characteristics of Study Population

A total of forty hyperthyroid subjects were studied. Females constituted a larger proportion, 32 (80%) of the studied subjects, while males were 8 (20%), giving a female to male ratio of 4 : 1 (F : M = 4 : 1). The mean age (SD) of the test subjects was 36.16 (8.43) years.

Eleven (27.5%) of these hyperthyroid subjects were treatment naive, while 29 (72.5%) were on antithyroid therapy. The total mean duration of thyroid disease in the entire studied population was 27.40 (23.6) months, while the mean duration of antithyroid therapy was 13.07 (19.3) months for the hyperthyroid subjects on treatment.

All the hyperthyroid subjects studied fulfilled criteria of overt hyperthyroidism as evidenced by their mean Wayne index score of 27.1 with a range of 22 to 41 and the corresponding thyroid function indices: mean FT4 39.44 (24.77) pmol/L, FT3 12.13 (7.83) pmol/L, and TSH 0.26 (0.03) *μ*/U/ml. There was, however, no significant difference in mean PTH levels between hyperthyroid cases and normal controls [5.2 (5.4) versus 6.3 (5.0) pmol/L, *p* = 0.41].

Comparison of some clinical parameters between hyperthyroid and controls showed a lower trend in body mass index for hyperthyroid subjects (24.40 (4.34) versus 26.32 (4.07), *p* = 0.06). However, among male patients, there was a significant reduction in mean BMI in hyperthyroid subjects compared to controls [21.40 (2.25) kg/m^2^ versus 27.5(4.88) kg/m^2^ (*p* = 0.004)]. The other clinical parameters showed no significant differences between the hyperthyroid and control subjects.

The bone markers osteocalcin, total alkaline phosphatase, and urinary calcium/creatinine ratio were significantly elevated in thyrotoxic subjects compared with controls (*p* < 0.001, *p* < 0.001, and *p* = 0.02, resp.), while the mean serum levels of 25-OHVit D, albumin-corrected calcium, PTH, and phosphate were comparable between the groups (*p* = 0.50, *p* = 0.31, *p* = 0.19, and *p* = 0.16, resp.).

### 3.2. Bone Densitometry

The ISCD *z*-score of the hyperthyroid subjects was significantly low compared to controls [−1.99 (1.15) versus 0.2 (1.09), *p* < 0.001]. Using the ISCD *z*-score criteria, 18 (45%) of the hyperthyroid subjects were diagnosed to have osteoporosis, while all the control subjects were free of osteoporosis. The mean ages of persons with and without osteoporosis were 38.7 (9.0) and 34.4 (7.7) years, respectively. There was no significant difference in the mean age between these two groups of hyperthyroid patients (*p* = 0.14). This study found no significant difference in the osteoporosis rate between treatment-naive hyperthyroid subjects and hyperthyroid subjects on treatment with sustained thyrotoxicosis [5/11 (54.5%) versus 12/29 (58.6%) *p* = 0.82].

There were significant differences in the mean BMD *z*-scores between hyperthyroid and normal controls [−0.15 (0.02), *p* < 0.001] and between osteoporosis-positive and -negative hyperthyroid subjects [−0.15 (0.02), *p* < 0.001] but not between treatment-receiving and treatment-naive hyperthyroid cases [0.005 (0.025), *p* = 0.84].

The BMD *z*-scores showed a significant negative correlation to FT4 (*r* = −0.33, *p* = 0.009), FT3 (*r* = −0.49, *p* = 0.005), and osteocalcin (*r* = −0.61, *p* < 0.001) but a positive correlation with thyroid-stimulating hormone (TSH) (*r* = 0.54, *p* < 0.001). There was also a direct correlation between the BMD *z*-scores and creatinine levels (*r* = 0.43, *p* = 0.001) and an inverse correlation between BMD *z*-scores and serum calcium levels (*r* = −0.27, *p* = 0.035), corrected calcium (*r* = −0.31, *p* = 0.039), and serum alkaline phosphatase (*r* = −0.53, *p* < 0.001). However, no correlation between BMD z-scores and serum phosphorus, 24 hour calcium excretion or phosphorus excretion was found. Furthermore, no correlation was established between BMD *z*-scores and serum 25-OHVit D, PTH, or any of the clinical parameters examined (age, BMI, waist circumference, duration of disease, and duration of antithyroid treatment) (Table 1).

**Figure 1:**
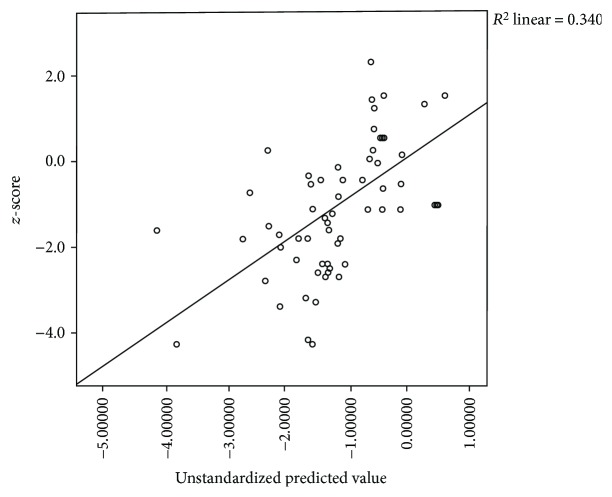
Scatter plot of multiple regressions. Predictors: TSH, FT4, and FT3. Dependent variable: BMD (*z*-score). *F*-statistics: level of significance = 13.068; <0.001. Unstandardized coefficients, B, and level of significance for FT3: −0.017; 0.087, FT4: −0.045; 0.137, TSH: 1.600; 0.015.

**Figure 2:**
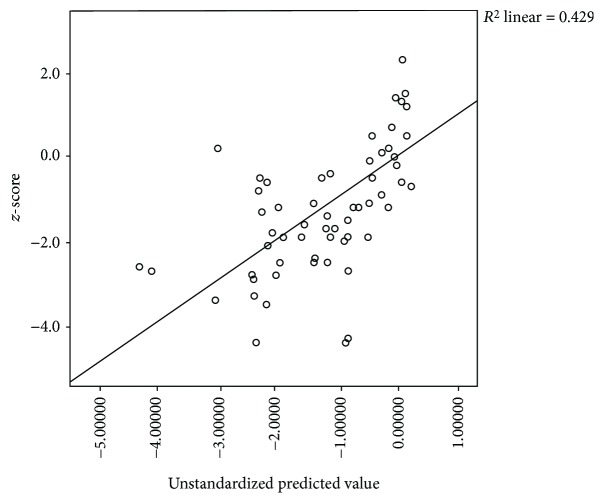
Scatter plot for multiple regressions. Predictors (bone markers): osteocalcin and alkaline phosphatase. Dependent variable: BMD (*z*-score). *F*-statistics: level of significance = 28.891; <0.001. Unstandardized coefficients, B, and level of significance for osteocalcin: −0.034; <0.001, alkaline phosphatase: −0.003; 0.007.

Multiple regression analysis showed that only osteocalcin (*p* = 0.000) and TSH (*p* = 0.015) were independently correlated to BMD *z*-score in our study subjects (Figures 1 and 2).

### 3.3. Clinical, Biochemical, and Hormonal Characteristics of Osteoporosis-Positive Compared to Osteoporosis-Negative Hyperthyroid Subjects

The clinical, biochemical, and hormonal characteristics of hyperthyroid subjects positive and negative to osteoporosis were compared. The results showed that, with the exception of proximal myopathy (*p* = 0.035), there were no significant differences in the other clinical characteristics, such as waist circumference, the presence of cramps/spasms, muscle weakness/numbness, abdominal pain, and nocturia between osteoporosis-positive and osteoporosis-negative hyperthyroid subjects (Table 2). There was a significant difference in BMI for males with positive and negative osteoporosis (*p* = 0.006) but not for females (0.753).

For the biochemical indices, a significant difference was observed in the serum alkaline phosphatase (*p* < 0.001) and urinary creatinine (*p* = 0.03) levels between osteoporosis-positive and osteoporosis-negative subjects. However, the levels of serum calcium, phosphate, creatinine, calcium, corrected calcium, and phosphate excretion were comparable between the two groups of hyperthyroid subjects (Table 3).

In addition, subjects with osteoporosis demonstrated significant differences in the levels of FT3 (*p* = 0.02), TSH (*p* = 0.006), and osteocalcin (*p* < 0.001) between osteoporosis-positive and -negative patients. However, there were no significant differences in the levels of 25-hydroxyvitamin D and parathyroid hormone between the two groups (Table 4).

## 4. Discussion

Bone health in hyperthyroidism is a poorly reported aspect of thyrotoxicosis especially in sub-Saharan Africa, probably because osteoporosis is considered a disease of developed nations and is considered to constitute fewer problems compared to devastating infectious diseases in the developing countries. Osteoporosis remains an unexplored terrain in Nigeria as dual-energy X-ray absorptiometry, the current gold standard for determining BMD, is not easily accessible in Nigeria.

The bone changes in hyperthyroidism are characterized by an enhanced bone turnover in both trabecular and cortical bone leading to increased porosity and mobilization of bone mineral [20]. Cortical bone is affected to a greater extent than trabecular bone. In line with the previous clinical investigation, BMD was measured at the distal radius (forearm) in hyperthyroidism in this study. It has been demonstrated that predominant bone loss was found in the forearm [21]. Jagoda et al. suggested that BMD is best measured at distal forearm in hyperthyroidism [22].

In addition, it has been reported that the distal radius DXA scan method also provides adequate accuracy for in vivo determination of spinal, femoral neck, and Ward's triangle osteoporosis [23] and the precision value for the distal radius is 1.9% [24].

The prevalence of osteoporosis in this present study was found to be high at a rate of 45%. This finding indicates that osteoporosis is a common occurrence in thyrotoxicosis among Nigerian patients with hyperthyroidism. Although, the 45% rate of osteoporosis found in our study is quite higher than those observed in Caucasians of 10–20% but lower than those in Asians [25–27]. It, however, contradicted Siddiqi et al.'s [28] study which did not document osteoporosis but reported osteopenia only among his studied population. Based on the aforementioned studies, it can be concluded that bone loss in hyperthyroidism is a common occurrence, even among Blacks of ages 21–50 years. This could be attributed to racial differences and probably severity of hyperthyroidism among our studied population.

Although osteoporosis is known to be common among the aging population, especially in the female gender [29]; however, in our cohort, overt hyperthyroidism resulted in osteoporosis regardless of age or gender. This might probably be due to racial differences observed between the cohorts.

Furthermore, with the varied clinical presentations of hyperthyroidism, an association between proximal myopathy and osteoporosis was established in this study. Therefore, the occurrence of proximal myopathy should raise a suspicion of metabolic bone disease.

Also, increased bone osteoblastic and osteoclastic activities were observed in this study resulting in an accelerated bone turnover in favor of bone resorption leading to low bone density. The relationship between biochemical parameters, bone turnover biomarker expressions, and BMD further accounted for bone loss that was demonstrated.

The mechanisms of bone loss can be attributed to the deleterious effect of excess circulating thyroid hormones and reduction of TSH on bone cells acting either singly or in synergy through the hypothalamic-pituitary-thyroid axis. This study confirmed the findings of previous studies [30, 31] which showed that free triiodothyronine has either a direct action on osteoblastic cells in vivo, which in turn mediate osteoclastic bone resorption or via direct action on osteoclastic cells via the existing thyroid receptors present on the bone cells. Bassett et al.'s study [31] confirmed that excess thyroid hormone rather than thyrotropin deficiency induces osteoporosis in hyperthyroidism.

The specific role of TSH as a mediator of bone loss has not yet been mutually established. While one study suggested a direct role for low TSH alone as a mediator of bone loss, another recommended indirect roles. Abe et al.'s study [32] demonstrated a direct role of TSH as a negative regulator of bone turnover by inhibiting the formation and survival of osteoclasts, as well as the inhibition of osteoblast differentiation and the expression of type 1 collagen expression through TSH receptors in osteoblasts and osteoclast cells. An indirect effect via TSH action was documented in a study by Morimura et al. [33] where the author observed an increased expression of type 2 iodothyronine deiodinase in human osteoblasts which was stimulated by thyrotropin (TSH). However, reduction in the levels of TSH in hyperthyroid state leads to a decrease expression of type 2 iodothyronine deiodinase enzyme culminating in high levels of triiodothyronine and thus stimulating bone resorption.

Our study confirmed previous studies that thyroid hormones and TSH play significant roles in the occurrence of osteoporosis in hyperthyroidism.

The limitation of this study is the failure to carry out immunological studies. However, we aim to carry out such studies in the near future. It is important to note that earlier studies using immunological parameters have not conclusively proven that the etiology of hyperthyroidism had any relevant impact on the severity of bone loss [34, 35].

## 5. Conclusion

It is imperative for clinicians to adopt a holistic approach to the management of patients particularly towards controlling TSH in hyperthyroidism in order to reduce bone loss as well as reducing the burden associated with undiagnosed bone complication, in view of the high prevalence of osteoporosis in hyperthyroid patients observed in this study.

## Figures and Tables

**Table 1:** Correlation of some variables with BMD *z*-scores.

Variable	Pearson's correlation	Level of significance
FT4	−0.33	0.009
FT3	−0.49	0.005
TSH	0.54	0.000
Corrected calcium	−0.31	0.039
PTH	0.14	0.206
Serum creatinine	0.43	0.001
Urinary calcium	−0.17	0.142
Urinary phosphate	−0.21	0.057
Urine creatinine	0.23	0.043
Osteocalcin	−0.61	0.000
Alkaline phosphatase	−0.53	0.000
Vitamin D	0.15	0.197
Age	0.16	0.166
BMI	0.25	>0.05

**Table 2:** Comparison of clinical characteristics between osteoporosis-positive and osteoporosis-negative hyperthyroid subjects.

Clinical features	Study subjects with osteoporosis	Study subjects without osteoporosis	*p* value
Body mass index (kg/m^2^)	24.56 (4.6)	22.71 (2.9)	0.16
Waist circumference (cm)	76.26 (9.53)	77.33 (10.45)	0.8
Cramps/spasms	11 (61.11%)	16 (72.73%)	0.08
Muscle weakness/pains	6 (33.33%)	7 (31.82%)	0.16
Proximal myopathy	9 (50.0%)	10 (45.45%)	0.035^∗^
Numbness	8 (44.44%)	12 (54.55%)	0.2
Abdominal pains	4 (22.22%)	(4) 18.18%	0.2
Nocturia	5 (27.78%)	4 (18.18%)	0.1

^∗^Significant *p* values. Results are expressed as percentage, mean, and standard deviation. Occurrence of proximal myopathy showed an association with osteoporosis.

**Table 3:** Comparison of biochemical parameters between osteoporosis-positive and osteoporosis-negative hyperthyroid subjects.

Biochemical indices	Study subjects with osteoporosis	Study subjects without osteoporosis	*p* value
Serum calcium (mmol/l)	2.3 (0.2)	2.2 **(**0.2)	0.3
Corrected calcium	2.30 (0.19)	2.29 (0.19)	0.31
Phosphorus (mmol/l)	1.74 (1.91)	1.33 (0.4)	0.16
Creatinine (*μ*mol/l)	64.18 (18.93)	67.84 (22.62)	0.56
Alkaline phosphatase (IU/l)	269.24 (197.61)	132.05 (78.04)	0.000^∗^
24 hour calcium excretion (mg/kg/day)	650.71 (676.80)	476.70 (268.34)	0.16
24 hour phosphorus excretion (mg/kg/day)	1796.88 (1996.9)	1164.12 (1064.16)	0.12
Urine creatinine (*μ*mol/l)	5891.76 (3001.62)	7483.09 (2308.05)	0.03^∗^

^∗^Significant *p* values. Results are expressed as a mean and standard deviation.

**Table 4:** Comparison of hormonal profiles between osteoporosis-positive and osteoporosis-negative hyperthyroid subjects.

Hormonal indices	Study subject with osteoporosis	Study subjects without osteoporosis	*p* value
Free thyroxine (pmol/L)	33.12 (21.80)	27.64 (24.40)	0.4
Free triiodothyronine (pmol/L)	12.72 (8.51)	7.81 (6.91)	0.02^∗^
Thyroid-stimulating hormone (*μ*/U/ml)	0.25 (0.03)	0.41 (0.25)	0.006^∗^
Osteocalcin (ng/ml)	52.79 (15.62)	29.85 (20.56)	0.000^∗^
25-Hydroxyvitamin D (nmol/l)	53.60 (15.77)	66.78 (16.25)	0.05
Parathyroid hormone (pmol/L)	5.43 (5.85)	5.57 (0.93)	0.90

^∗^Significant *p* values. Results are expressed as a mean and standard deviation.
